# Engineering the Composition of Microfibers to Enhance the Remodeling of a Cell-Free Vascular Graft

**DOI:** 10.3390/nano11061613

**Published:** 2021-06-20

**Authors:** Fang Huang, Yu-Fang Hsieh, Xuefeng Qiu, Shyam Patel, Song Li

**Affiliations:** 1 UC Berkeley & UCSF Joint Graduate Program in Bioengineering, Berkeley & San Francisco, CA 94720 & 94143, USA; zoeyfhuang@berkeley.edu; 2 Department of Bioengineering, University of California, Berkeley, CA 94720, USA; r96223168@berkeley.edu (Y.-F.H.); qxf027@163.com (X.Q.); shyampatel@berkeley.edu (S.P.); 3 Department of Bioengineering and Department of Medicine, University of California, Los Angeles, CA 90095, USA

**Keywords:** vascular graft, microporosity, SDF-1α, tissue engineering

## Abstract

The remodeling of vascular grafts is critical for blood vessel regeneration. However, most scaffold materials have limited cell infiltration. In this study, we designed and fabricated a scaffold that incorporates a fast-degrading polymer polydioxanone (PDO) into the microfibrous structure by means of electrospinning technology. Blending PDO with base polymer decreases the density of electrospun microfibers yet did not compromise the mechanical and structural properties of the scaffold, and effectively enhanced cell infiltration. We then used this technique to fabricate a tubular scaffold with heparin conjugated to the surface to suppress thrombosis, and the construct was implanted into the carotid artery as a vascular graft in animal studies. This graft significantly promoted cell infiltration, and the biochemical cues such as immobilized stromal cell-derived factor-1α further enhanced cell recruitment and the long-term patency of the grafts. This work provides an approach to optimize the microfeatures of vascular grafts, and will have broad applications in scaffold design and fabrication for regenerative engineering.

## 1. Introduction

Tissue engineering is a fast-growing field centered on creating equivalents to native tissue for the replacement of trauma- or disease-damaged tissue or organs [1,2,3,4]. In particular, there is a great need for the replacement of obstructed blood vessels. Every year in the USA, over 500,000 vascular grafts are used in bypass procedures for coronary arteries and peripheral arteries. Most of the vascular grafts used are autologous grafts [5]. An autologous graft has the advantages of being native and biocompatible to the patient, free of immune rejection, and having optimal biological, structural, and mechanical properties. However, the use of autologous grafts is limited by their availability: one-third of patients with peripheral arterial disease do not have suitable autologous grafts [6,7,8]. Moreover, harvesting autologous grafts requires additional surgeries and may potentially lead to donor site morbidity. When availability limits the use of autologous grafts, artificial synthetic vascular grafts are used as an alternative. Many commercial vascular grafts made with inert materials have been developed over the past decade, such as Dacron (polyethylene terephthalate; PET) and Teflon (expanded polytetrafluoroethylene; ePTFE). However, due to their surface thrombogenicity, suboptimal elasticity and compliance, these vascular grafts are prone to acute thrombus formation and intimal hyperplasia, resulting in an unsatisfactory in vivo performance in terms of patency, despite the presence of heparin coating [9,10,11]. Therefore, these vascular grafts are limited to large-caliber vessels and the development of successful small-diameter vascular grafts (<5 mm) remains challenging. An ideal graft would provide the necessary mechanical strength to withstand arterial pressure, anti-thrombogenic surface to maintain patency, and biochemical cues for the recruitment and infiltration of appropriate cell types, as well as being able to degrade and be cleared from the system in a timely manner when the vascular regeneration is completed. However, tissue engineered grafts in general, including synthetic and decellularized native arteries, have limited host cell infiltration and remodeling even 6–12 months after implantation [12,13,14,15,16]. Therefore, the design and fabrication of scaffolds with appropriate microstructure and porosity to promote cell infiltration and vascular remodeling are critical.

In addition to microstructure of the scaffolds, biochemical cues that can promote cell recruitment and enhance cell functions will facilitate graft remodeling. For example, stromal cell-derived factor-1α (SDF-1α) is a chemokine that attracts a variety of CXCR4^+^ and CXCR7^+^ cells [17,18,19], including progenitors of endothelial cells (ECs) and smooth muscle cells (SMCs), which are essential for blood vessel regeneration and angiogenesis. Furthermore, heparin, an anticoagulant commonly used in vascular surgeries, can bind to SDF-1α with high-binding affinity [20,21]. It was also shown that binding to heparin stabilizes and protects SDF-1α from enzymatic degradation [22]. Therefore, SDF-1α can be easily immobilized onto heparin-coated vascular grafts to accelerate *in situ* vascular regeneration [16].

In this study, we engineered the degradation of microfibers by introducing a fast-degrading polymer to promote cell infiltration into the scaffold. The tubular scaffold, in combination with the immobilization of heparin and SDF-1α on the surface, enhanced the remodeling of the blood vessel wall.

## 2. Materials and Methods

### 2.1. Fabrication of Vascular Grafts 

**Figure 1:**
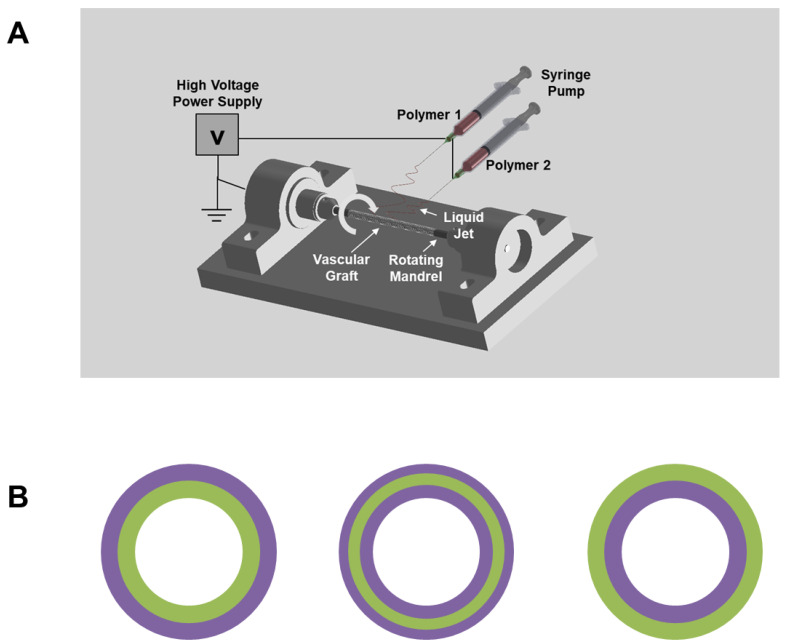
(**A**) Set-up of electrospinning of the vascular graft. Generally, a single polymer solution was eluted for multi-layered and single-layered vascular grafts, with the exception of co-electrospinning, in which two polymer solutions were eluted simultaneously. (**B**) Schematic design of multi-layered grafts, where PLCL is indicated by green color and PDO is indicated by purple color.

Electrospinning was performed to fabricate vascular grafts with a customized electrospinner set-up (Figure 1A). Three different formulations of polymer solution were used in this study to fabricate four single-layered and three multi-layered vascular grafts: (1) the Poly(L-lactide-co-ε-caprolactone (PLCL) (70% L-lactide and 30% ε-caprolactone by weight) solution was produced by dissolving 15% PLCL (weight by volume (w v^−1^)), 1% poly(propylene glycol) (volume by volume) in highly volatile Tris-hexafluoro-2-propanol (HFIP), which contained 0.05% w v^−1^ Tris-HCl; (2) the Polydioxanone (PDO) solution was produced by dissolving 20% PDO (w v^−1^) in Tris-HFIP; (3) the PLCL/PDO blend solution was produced by mixing the aforementioned PLCL and PDO solution at a 1:1 ratio by volume.

To produce single-layered vascular grafts, the three aforementioned polymer solutions were independently used to spin PLCL, PDO, and PLCL/PDO blend grafts. The fourth single-layered vascular graft was manufactured by co-electrospinning a 15% PLCL and a 20% PDO solution as follows: two syringes containing 15% PLCL and 20% PDO solution were attached to the same syringe pump so that their polymer solution dispensing rates were identical. Two silicone tubes were separately connected to the two syringes. The polymer solutions were eluted from two different needles that were both secured to the same spinneret. The resulting electrospun fibers were collected onto the same mandrel and allowed to be air dried overnight under a laboratory fume hood to remove residual solvent. The aforementioned four single-layered vascular grafts will be referred to as PLCL, PDO, PLCL/PDO blend, and PLCL/PDO co-spun, respectively.

To produce multi-layered vascular grafts, 15% PLCL and 20% PDO polymer solutions were used. Only one polymer solution was allowed to spin at any one time. One solution was allowed to electrospin for a certain amount of time until the graft wall reached the desired thickness; at this point, the solution was immediately switched to the other formulation, and spinning was allowed to continue until the final thickness reached approximately 200–250 μm. The three structures manufactured in this study are as follows: (1) PLCL as luminal layer (~50 μm) and PDO as exterior layer; (2) PDO as luminal layer and PLCL as exterior layer (~50 μm); (3) PLCL as middle layer (~50 μm) and PDO as both luminal and exterior layer (Figure 1B).

The aforementioned three multi-layered vascular grafts will be referred to as PLCL-Lumen, PLCL-Exterior, and PLCL-Middle, respectively.

### 2.2. Heparin Conjugation and Quantification of Heparin Activity

A two-stage (ammonia followed by hydrogen) plasma treatment was used to introduce amine functional groups on the surface of the PLCL/PDO blend grafts [23,24]. The untreated and plasma-treated grafts were incubated with a heparin solution (Sigma Aldrich) in cyanoborohydride coupling buffer overnight. The anti-thrombogenic activity of heparin was quantified using the Anti-thrombin-III assay. In brief, control and heparinized grafts were incubated in diH_2_O for 1 and 7 days at room temperature on a shaker. At the end of day 1 and 7, the activity of the heparin remaining on the grafts was quantified with the Anti-thrombin-III assay following a previously described protocol [23].

### 2.3. Mechanical Characterization of Vascular Grafts

The overall fibrous structure of electrospun vascular grafts was examined and imaged using a scanning electron microscope (SEM) (Hitachi TM-1000). In addition, to determine the mechanical properties of vascular grafts, graft ring segments of 1 mm in diameter and 2 mm in longitudinal width were prepared and subjected to uniaxial tensile testing in the radial direction. In detail, two 0.3-mm-diameter stainless steel wires (McMaster-Carr) were inserted through the lumen of the ring segment and fixed on mechanical loading grips. The ring segment was deformed until failure as the two wires were pulled in opposite uniaxial directions at a rate of 0.1 mm sec^−1^. The applied force and corresponding deformation were recorded. The Young’s modulus was calculated based on the applied force, deformation, and dimensions (thickness and longitudinal width) of the graft ring segments. The peak stress prior to failure was noted as the ultimate tensile strength (UTS). 

### 2.4. Hydrolytical Degradation of Vascular Grafts

Degradation of untreated and heparinized vascular grafts caused by hydrolysis was measured by mass loss. The untreated and heparinized PLCL/PDO blend grafts were cut into 1-cm long conduits. The weight of each conduit sample was measured before the degradation assay. To degrade the grafts, the conduit samples were placed in excess phosphate-buffered saline (PBS). During the degradation study, the samples were kept at 37 °C on a shaker maintained at 120 rpm to mimic the physiological flow condition. To avoid random local hydrolytic degradation resulting from acidic polymer remnants, PBS was used in an excess amount and changed daily throughout the study. Samples were collected after 7, 14, 21, and 28 days of degradation. The samples were washed with water to remove residual salt particles and vacuum dried overnight. The weight of the samples at each time point was measured using a precision scale. The percentage degraded was calculated using Equation (1):

(1) $$\%\;Degradation=\frac{w_{0}-w_{t}}{w_{0}}$$

In addition, the UTS of post-degraded samples was measured at each time point.

### 2.5. SDF-1α Immobilization and In Vitro Release

To immobilize SDF-1α onto the grafts, heparinized grafts were incubated with SDF-1α (500 ng mL^−1^) in PBS overnight at 4 °C to foment its binding to heparin and, hence, its immobilization onto the grafts. To quantify the amount of SDF-1α initially loaded onto the grafts, the grafts were removed from the solution, and an enzyme-linked immunosorbent assay (ELISA) was performed to measure the concentration of unbound SDF-1α. The amount of SDF-1α immobilized onto the graft was calculated based on the concentration of initial and unbound SDF-1α. The plasma-treated blend vascular graft was used as a control to measure the amount of SDF-1α passively absorbed onto the grafts. 

The *in vitro* release of immobilized SDF-1α was evaluated over a period of 7 days. The SDF-1α immobilized heparinized grafts were removed from SDF-1α solution and placed in 1 mL of PBS. The grafts were kept at 37 °C in an incubator. At the end of 1, 4, and 7 days of incubation, the grafts were removed from the solution, and the amount of released SDF-1α in the supernatant was quantified using an ELISA kit. 

### 2.6. In Vivo Subcutaneous Implantation and Explantation of the Scaffolds

The animal protocol was developed and approved before surgery. All procedures were approved by the Institutional Review Board Service and the Institutional Animal Care and Use Committee at the University of California, Berkeley (protocol code R300-0915B approved on February 18, 2015). Two-month-old male Sprague-Dawley (SD) rats (approximately 260 g) were purchased from the Charles River animal facility. The rats were anesthetized with 2.0% isoflurane in 70% nitrous oxide and 30% oxygen. Abdominal skin was dissected; untreated single-layered vascular grafts (PLCL, PDO, PLCL/PDO blend, and PLCL/PDO co-spun) were placed in between fascia and muscle without being sutured to surrounding tissue. The wound was sutured with a 4-0 needle. At 2 weeks after implantation, the animals were euthanized, and the vascular grafts were explanted.

### 2.7. In Vivo Bypass Implantation and Explantation of Vascular Grafts

The left common carotid artery was dissected, clamped, and transected. The heparinized vascular graft, with and without SDF-1α, was sutured end to end using a 9-0 needle. Immediately after implantation, suture holding strength was assessed by examining the suture sites for leakage. A number of arterial pulses were observed to ensure the vascular grafts were able to provide adequate tensile and recoil strength. To determine the long-term patency of the graft at the time of explantation after 2 months in vivo, the blood flow in the blood vessel at the distal end of the graft, in the live animal under anesthesia, was examined. To elaborate, forceps were used to hold the distal blood vessel near the anastomotic site while the distally attached native carotid artery was dissected. The forceps were subsequently released to determine whether unobstructed blood flow was passing through the graft. The graft was defined as being patent only if blood flow and noticeable pulsation were observed through the graft. Lastly, the animals were euthanized. The vascular grafts were explanted and prepared for histological analysis, immunohistochemistry, and *en face* immunofluorescence staining. 

### 2.8. Histological Analysis

The grafts were explanted and fixed with 4% paraformaldehyde (PFA) for 2 h and dehydrated in 30% sucrose overnight at 4 °C. The dehydrated grafts were frozen in an optimal cutting temperature compound (Tissue-Tek), and sectioned into 10 μm-thick slices using a cryostat. Hematoxylin and Eosin (H&E) staining was performed to determine the degree of cell penetration into the grafts. Slides were fixed again with 4% PFA for 10 min. Fixed slides were washed with water for 5 min and followed by hematoxylin staining for 15 min. Slides were quickly rinsed with water and stained with eosin for 1 min. Stained slides were dehydrated for 2 min each with increasing concentrations of ethanol (70%, 85%, and 100% ethanol) and followed by Xylene for 5 min to clear residual ethanol. The finished slides were air-dried and mounted with xylene-based Permount medium.

### 2.9. Immunohistochemistry

Sectioned samples were fixed with 4% PFA for 15 min, followed by permeabilization with 0.5% triton-100 for 10 min. The slides were blocked with 4% donkey serum in PBS for an hour and incubated overnight at 4 °C with the primary antibodies against EC/endothelial progenitor cell (EPC) markers CD31 and CD34; the SMC markers α-Smooth muscle actin (αSMA) and calponin 1 (CNN1); and the immune cell markers CD68, CD163, and CD11b (Table 1). The samples were then washed with PBS and incubated with Alexa-Fluor 488 or Alexa-Fluor 546 labeled secondary antibodies and Hoechst 33342, followed by fluorescent microscopy.

### 2.10. En Face Immunofluorescence Staining

Each explanted graft was cut into 4 slices along the longitudinal direction using microscissors. The samples were fixed with 4% PFA for 30 min, washed with PBS, and blocked with 4% donkey serum for an hour. Samples were incubated with primary antibodies against EPC marker CD34 and EC marker CD31. The samples were then washed with PBS and incubated with Alexa-Fluor 488 or Alexa-Fluor 546 labeled secondary antibodies and Hoechst 33342, followed by confocal microscopy.

### 2.11. Cell Number Quantification

ImageJ was used to conduct quantitative analysis of infiltrated cell numbers. Images of vascular grafts stained with Hoechst 33342 were imported to ImageJ and analyzed with the cell counter plugin.

### 2.12. Statistical Analysis

Student’s *t*-test was performed to detect whether a significant difference exists between two sample groups. For multiple sample comparison, analysis of variance (ANOVA) was used first to detect whether or not there was a significant difference among the groups; Holm’s *t*-test was subsequently performed on all possible pairs to isolate the different samples. A *p*-value of 0.05 or less was considered to have a significant difference.

## 3. Results

### 3.1. PDO as a Fast-Degrading Polymer for Vascular Graft Fabrication

**Figure 2:**
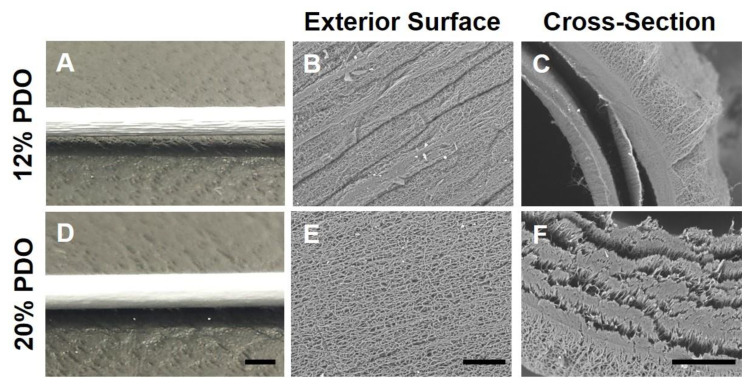
Characterization of electrospun microfibrous PDO vascular grafts. Gross structure (**A**,**D**, scale bar = 1 mm) and SEM images of microstructure of exterior (**B**,**E**, scale bar = 100 μm) and cross-section (**C**,**F**, scale bar = 100 μm) surface of PDO vascular grafts electrospun at 12% and 20% by weight.

The electrospinning technique was used to fabricate microfibrous tubular grafts (Figure 1A). PDO is a fast-degrading polymer. Our preliminary data (not shown) demonstrated that electrospun PDO sheet lost its mechanical strength drastically *in vitro* after a month, suggesting it degrades rapidly and could potentially serve as the sole graft material or as an additive to PLCL vascular graft to promote cell infiltration. Initial attempts to electrospin 12% PDO in Tris-HFIP (w v^−1^) resulted in grafts with rough exterior surfaces (Figure 2). Moreover, 12% PDO resulted in severe delamination. The issues of rough surface and delamination were not alleviated by adjusting the electrospinning parameters such as voltage, flow rate of the polymer solution, and mandrel–needle distance. To resolve these issues, we increased the concentration of the polymer solution. We electrospun PDO grafts with a gradient of polymer concentration, ranging from 12% to 20%. Higher concentrations of PDO (20%) resulted in smoother graft surfaces. The SEM images of the cross-section of the 20% PDO grafts (Figure 2) showed that its structural integrity was superior to 12% PDO grafts, yet there was partial delamination. Although the weaker structural integrity of PDO suggests its mechanical integrity might need improvement, it also led to a more porous structure, which is potentially beneficial for cell infiltration.

### 3.2. Using PDO to Produce Fast-Degrading Layer(s) of Vascular Grafts

PLCL is an elastic and slow-degrading polymer, but electrospun PLCL grafts had low porosity and cell infiltration. Therefore, we investigated the possibility of using PDO as fast-degrading layer(s) in the fabrication of PLCL grafts: (1) PLCL as the luminal layer (~50 μm) and PDO as the exterior layer; (2) PDO as the luminal layer and PLCL as the exterior layer (~50 μm); (3) PLCL as the middle layer (~50 μm) and PDO as both the luminal and the exterior layer (Figure 1B). 

**Figure 3:**
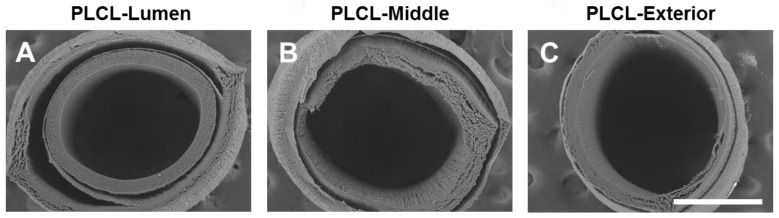
Characterization of multi-layered vascular grafts. Cross-section surfaces of multi-layered vascular grafts with the following structure: (**A**) PLCL as the luminal layer and PDO as the exterior layer; (**B**) PLCL as the middle layer and PDO as both the luminal and the exterior layer; and (**C**) PDO as the luminal layer and PLCL as the exterior layer. Scale bar = 500 μm.

The SEM images of their cross-sections showed that all of the three formulas exhibited severe delamination, which is possibly explained by the weak intermolecular adhesion between PDO and PLCL. Since the integrity of the vascular graft is a basic requirement for it to provide the necessary mechanical and structural support, this severe delamination disqualified potential use of these multi-layered scaffolds as vascular grafts (Figure 3).

### 3.3. Making Single-Layer Vascular Grafts by Blending or Co-Electrospinning PLCL and PDO

**Figure 4:**
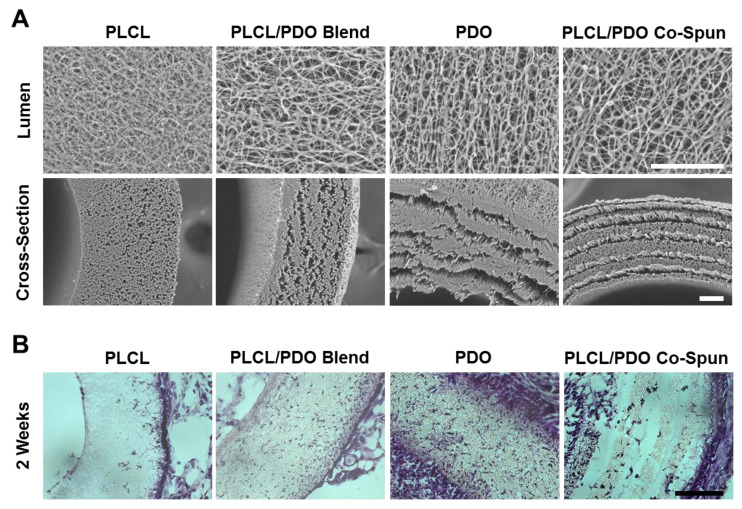
Characterization and in vivo cell infiltration of the single-layered microfibrous vascular grafts. (**A**) Luminal (**top**) and cross-section (**bottom**) surface of single-layered vascular grafts electrospun with 15% PLCL, PLCL/PDO blend, 20% PDO, and PLCL/PDO co-spun. Scale bar = 50 μm. (**B**) H&E staining of single-layered vascular grafts electrospun with 15% PLCL, 20% PDO, PLCL/PDO blend, as well as vascular grafts co-electrospun with PLCL and PDO at 2 weeks after subcutaneous implantation. Scale Bar = 100 μm.

We then explored the feasibility of using PDO as a fast-degrading polymer component for graft fabrication. In addition to 15% PLCL and 20% PDO grafts, two other single-layered vascular grafts were fabricated: (1) 15% PLCL and 20% PDO solution was thoroughly mixed at a 1:1 ratio and the mixed solution was electrospun to produce PLCL/PDO blend grafts; (2) the PLCL/PDO co-spun grafts were fabricated by co-electrospinning 15% PLCL solution and 20% PDO solution using two parallel needles simultaneously. SEM images showed that the fibers had diameters varying from the nano- to the micro-scale (Figure 4A). The luminal surfaces of the vascular grafts showed that 20% PDO, PLCL/PDO blend, and PLCL/PDO co-spun grafts had comparable morphology, and they all had larger fiber spacing and thicker fibers compared to 15% PLCL grafts. The SEM images of cross-sections of the vascular grafts showed that similarly to PLCL grafts, the blend grafts had organized morphology and structural integrity, but they possessed a more porous structure than PLCL, which was similar to that of the 20% PDO grafts. Interestingly, PLCL/PDO co-spun grafts had a non-delaminated “stacked” structure. Since the two polymers were not labeled, it is difficult to conclude the exact distribution of the two fibers in the graft. However, from the electrospinning setup and the morphology of the cross-section, we could postulate that the co-spun vascular graft consists of alternating layers of PLCL and PDO. Since the two syringes eluting PLCL and PDO solutions were positioned slightly above and below the rotating mandrel, the mandrel would consistently collect one polymer followed by the second, resulting in alternating layers. The narrow gaps between the layers are possibly due to the weak interactions between PLCL and PDO fibers. These results demonstrated that PDO grafts, either used alone or combine with PLCL, exhibit a more porous structure compared to PLCL grafts. This porous structure would potentially promote initial cell infiltration prior to the initiation of graft degradation.

### 3.4. Effects of Fast-Degrading PDO on In Vivo Cell Infiltration

To investigate the effects of PDO on in vivo cell infiltration into the grafts, the four single-layered vascular grafts (15% PLCL, 20% PDO, 15%PLCL/20%PDO blend, and 15%PLCL/20%PDO co-spun) were implanted subcutaneously into 2-month-old male SD rats. The grafts were explanted 2 weeks after implantation. H&E and Hoechst 33342 staining were performed to inspect cell infiltration. The results showed that at 2 weeks, all implanted grafts had cells infiltrated at different degrees (Figure 4B). The 15% PLCL grafts had the least cell infiltration, with the majority of the cells residing at the exterior rim of the grafts and few cells in the middle of the grafts. The 20% PDO grafts showed the highest number of cells residing throughout the grafts. The PLCL/PDO blend grafts also had significantly higher numbers of cells than the PLCL grafts. In contrast, PLCL/PDO co-spun grafts showed a unique cell distribution pattern in partially detached layers, likely due to the “stacked” fiber structure and the partial degradation of PDO. ImageJ was used to assess cell infiltration quantitatively from Hoechst 33342 staining with a cell counter plugin. The ratio of the number of infiltrated cells within PLCL, PLCL/PDO blend, PDO, and PLCL/PDO co-spun grafts was approximately 1:5:8:2, demonstrating a better cell infiltration into the PLCL/PDO blend and PDO grafts over the PLCL or PLCL/PDO co-spun grafts.

In addition to cell infiltration into the grafts, there were obvious differences for the capsule tissue formation around the implanted grafts. The 15% PLCL and PLCL/PDO blend grafts were relatively clean, with minimal capsule tissue growing around the grafts. The PLCL/PDO co-spun grafts had moderate amounts of surrounding tissue. In contrast, the 20% PDO grafts triggered a strong cellular response with the thickest layer of capsule tissue that confined the grafts.

Based on the results of cell infiltration, capsule tissue formation and structure integrity, we chose the PLCL/PDO blend grafts for subsequent studies on surface modification and bypass implantation.

### 3.5. Anti-Thrombogenic Modification of Microfibrous Vascular Grafts

**Figure 5:**
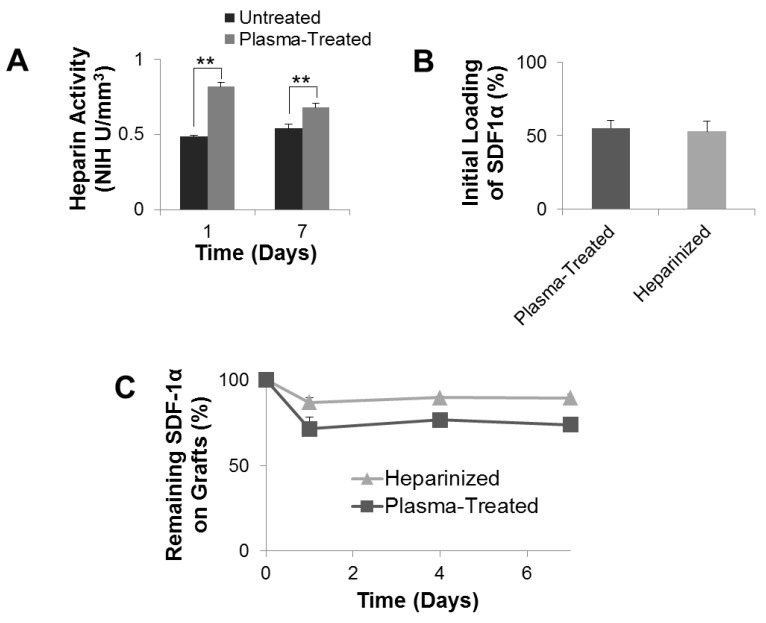
Quantification of heparin activity, SDF-1α initial loading, and release. (**A**) Activity of heparin passively absorbed onto untreated and heparin conjugated plasma-treated vascular grafts. (**B**) Initial loading of SDF-1α immobilized onto plasma-treated and heparinized vascular grafts. (**C**) The time course of SDF-1α release from plasma-treated and heparinized grafts. ** *p* < 0.01, (*n* = 3).

Electrospun vascular grafts underwent a two-stage ammonia plasma treatment. Such two-stage ammonia plasma treatment would introduce free amine functional groups onto the vascular graft surface, which were subsequently used to covalently conjugate heparin. Heparin is able to interact with anti-thrombin-III to prevent thrombosis [25,26]. Consequently, conjugation of anticoagulant heparin is believed to improve the blood compatibility of the vascular grafts [27]. Conjugated heparin was confirmed to be active and its activity was quantified with the Anti-thrombin-III assay. To study the stability of conjugated heparin, heparinized grafts and control grafts (with a coating of heparin by adsorption) were incubated in diH_2_O for up to 7 days. Quantification of Heparin activity with the Anti-thrombin-III assay showed that after 1 day of incubation in diH_2_O, the amount of active heparin remaining on the control graft was 0.48 NIH U mm^−3^, significantly lower than the heparinized graft, which had 0.851 NIH U mm^−3^ of active heparin (Figure 5A). This significance held for 7 days post-heparinization. At day 7, the control grafts possessed 0.54 NIH U mm^−3^ active heparin, significantly lower than the heparinized grafts, which had active heparin at 0.706 NIH U mm^−3^. This indicates that conjugated heparin was more stable than heparin that was passively absorbed onto the control grafts.

### 3.6. Mechanical Characterization and Degradation of the Grafts

**Figure 6:**
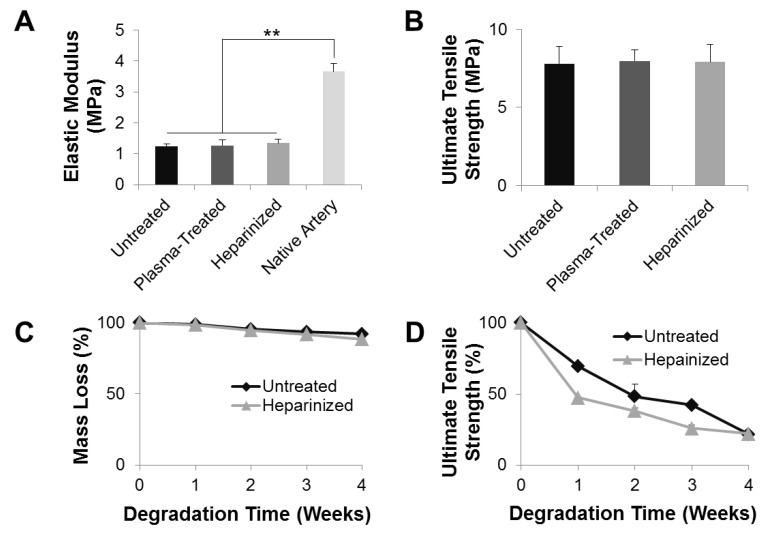
Mechanical and degradation characterization of microfibrous vascular grafts. (**A**) Elastic moduli of untreated, plasma-treated, heparinized vascular grafts, and native common carotid arteries. (**B**) UTS of untreated, plasma-treated, and heparinized vascular grafts. (**C**) Mass loss of untreated and heparinized vascular grafts during 4 weeks of hydrolysis in PBS under constant agitation. (**D**) Changes in the UTS of untreated and heparinized vascular grafts during 4 weeks of hydrolysis in PBS under constant agitation. ** *p* < 0.01, (*n* = 3).

To characterize the mechanical properties of the vascular grafts, a uniaxial tensile test in the radial direction was performed on the grafts. The plasma-untreated grafts had an elastic modulus of 1.24 ± 0.08 MPa (Figure 6A), which was at the same order of magnitude as native carotid arteries (3.66 ± 0.25 MPa). This result indicates that the synthesized vascular graft was able to provide sufficient arterial compliance immediately after implantation and was sufficient as an artery replacement. Neither the ammonia plasma treatment nor heparinization significantly altered the elastic modulus of the grafts: the elastic moduli of plasma-treated and heparinized grafts were 1.26 ± 0.19 and 1.34 ± 0.13 MPa, respectively. In addition, the untreated, plasma-treated, and heparinized grafts had comparable UTS of 7.81 ± 1.11, 7.98 ± 0.71, and 7.90 ± 1.13 MPa, respectively, with no significant differences (Figure 6B). These data demonstrated that the vascular grafts experienced no significant change of their mechanical properties during the process of ammonia plasma treatment or heparinization. 

Mass loss and UTS loss were studied over 4 weeks of *in vitro* hydrolytic degradation, which was carried out in PBS at 37 °C on a shaker maintained at 120 rpm to mimic the physiological in vivo flow condition. The plasma-untreated PLCL/PDO blend grafts lost approximately 8% of their original mass over the 4-week period in PBS (Figure 6C). However, their mechanical properties changed drastically: the UTS of untreated grafts was only 20% of their pre-degraded counterparts (Figure 6D). Heparinization facilitated water penetration into the grafts, and made the grafts more prone to hydrolysis. After 4 weeks of degradation, the heparinized grafts lost approximately 12% of their original mass and 80% of their UTS. Moreover, at any point throughout the degradation, heparinized grafts had less mass remaining and weaker UTS compared to untreated grafts. Although the loss in UTS is not desirable as UTS correlates with burst strength, these results indicate that heparinization facilitated degradation of the vascular graft which, in turn, could promote cell infiltration and tissue integration. Cell infiltration and tissue integration are necessary for tissue regeneration and would strengthen the vascular graft and offset the UTS loss during the degradation.

### 3.7. SDF-1α Immobilization for Cell Recruitment

Heparin is able to bind and stabilize SDF-1α [20,21,22,28]. To immobilize SDF-1α onto the grafts, heparinized grafts were incubated overnight with SDF-1α (500 ng mL^−1^) in PBS. Quantification by ELISA showed that the initial loading of SDF-1α onto heparinized grafts by SDF-1α-heparin interaction, or plasma-treated grafts by passive absorption, were comparable: at 52.78 ± 7.22% and 55.05 ± 5.36%, respectively (Figure 5B). The stability and *in vitro* release of SDF-1α were also examined (Figure 5C). The SDF-1α immobilized on heparinized grafts was stable and released relatively slowly. It had a burst release over the first 24 h and a steady release afterward. After 7 days of release, there was approximately 90% of the initially immobilized SDF-1α remaining on the heparinized graft. In comparison, the SDF-1α passively absorbed onto the plasma-treated grafts was less stable and released relatively rapidly. Approximately 70% of initial passively absorbed SDF-1α remained on the plasma-treated graft after 7 days of *in vitro* release. These results suggested that heparin conjugation onto plasma-treated grafts can prolong the lingering of SDF-1α, which would otherwise have a rapid clearance, at targeted sites.

### 3.8. In Vivo Performance and Long-Term Patency of Vascular Grafts

To evaluate the performance of PLCL/PDO blend grafts and the effect of SDF-1α in vivo, heparinized grafts with and without immobilized SDF-1α were implanted into the left common carotid artery of 2-month-old male SD rats by anastomosis. The implanted grafts were 1-cm long with an inner diameter of 1 mm. Following implantation, the vascular grafts were immediately functional. The grafts were able to hold the suture as the conduit showed no rupture or leakage at the suture sites. Grafts were examined 2 weeks and 2 months after implantation. Five animals were used per group per time point (*n* = 5 × 4 = 20). 

**Figure 7:**
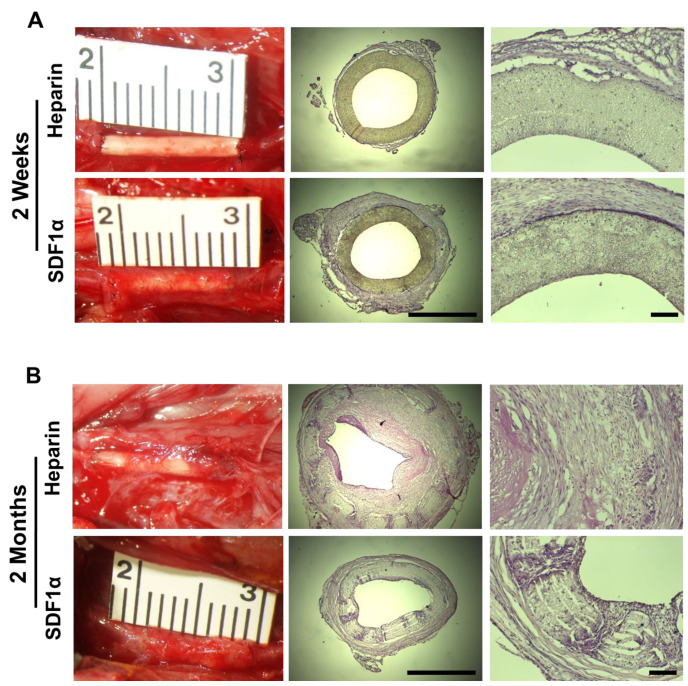
Tissue ingrowth and cell infiltration of vascular grafts. Vascular grafts explanted at (**A**) 2 weeks and (**B**) 2 months for the heparinized (**top**) and SDF-1α immobilized (**bottom**) group. Representative images of grafts *in situ* taken immediately prior to explantation (**left**) and H&E staining of the cross-section of explanted grafts at low (**middle**, scale bar = 1 mm) and high (**right**, scale bar = 100 μm) magnification.

Two weeks after implantation, micro-vessels were observed in the tissue surrounding the grafts for both the heparin and heparin-SDF-1α groups (Figure 7A). There was evidence of greater growth of surrounding tissues around the heparin-SDF-1α grafts, indicating more cell recruitment for graft remodeling than heparinized grafts. Despite the tissue growth on the exterior surface, H&E staining demonstrated that there was little cell infiltration into the grafts for both groups at 2 weeks, suggesting a different microenvironment from subcutaneous tissue. All patent grafts had a wide opening lumen with a layer of cells adhering onto the luminal surfaces that had potentially migrated from the adjacent blood vessels or been recruited from the streaming blood. Immunofluorescent staining of the occluded grafts showed that the cells in the clogs were CD45^+^, a marker of inflammatory cells, suggesting that thrombosis was the primary reason jeopardizing the patency of the vascular grafts at an early stage (data not shown).

Two months after implantation, PLCL/PDO blend grafts had partially degraded and were replaced by infiltrated cells for both the heparin and heparin-SDF-1α groups. Vascular grafts from both the heparin and heparin-SDF-1α groups had integrated further into the native tissue (Figure 7B). The amount of tissue growing around the grafts increased compared to their respective 2-week samples. Between the two groups, more tissue growth was observed on the heparin-SDF-1α grafts. There was also extensive cell infiltration into the wall of the grafts. Patency study showed that 20% (1 of 5) of heparinized grafts and 80% (4 of 5) of SDF-1α immobilized grafts were patent at 2 months after implantation, suggesting that SDF-1α immobilization improves long-term patency. The H&E staining demonstrated that the sole patent heparinized graft had a narrowed lumen, which was caused by extensive neointimal formation, suggesting that neointimal hyperplasia could be the major mechanism for vascular graft occlusion in the long term. In contrast, a thin layer of neointima formed within SDF-1α immobilized grafts without showing noticeable lumen narrowing. The diameter of heparin-SDF-1α grafts at 2 months was approximately 1 mm, comparable to the 2-week samples, demonstrating SDF-1α immobilization could impede neointimal hyperplasia. These data suggested that SDF-1α immobilization improves the overall performance of the vascular grafts. 

### 3.9. Recruitment of SMCs and Vascular Wall Remodeling

To obtain the desired mechanical properties, remodeling of the vascular wall relied on the successful recruitment and infiltration of cells. To investigate the recruitment of SMCs, which is the predominant cell type in the tunica media layer of the blood vessel, to the grafts, cross-section immunostaining with primary antibodies against αSMA, an early SMC marker, and CNN1, an intermediate SMC marker, was performed.

**Figure 8:**
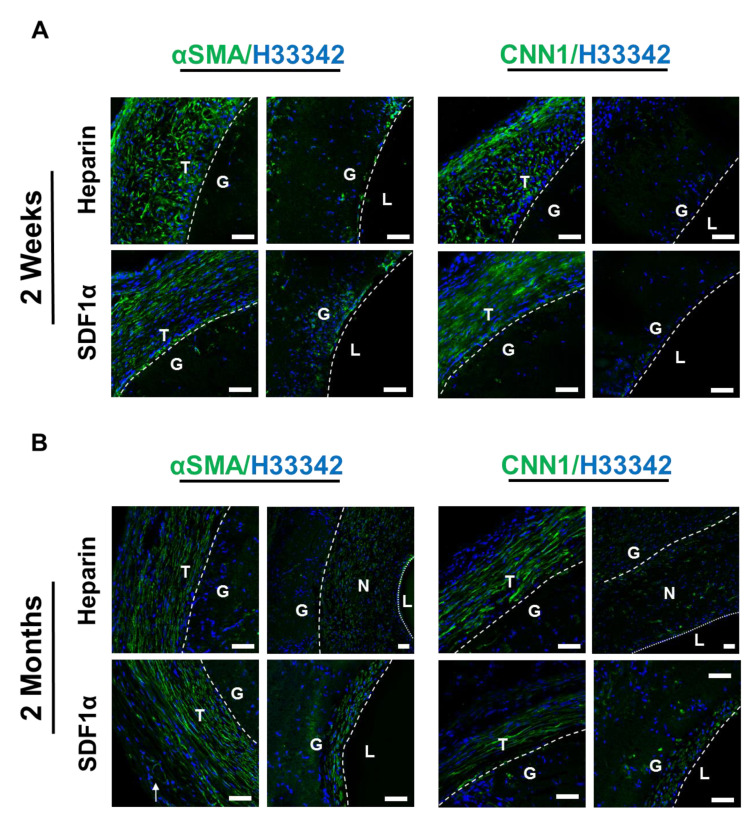
Identification and characterization of SMCs. Immunostaining for αSMA (**green**) and CNN1 (**green**) of heparinized and SDF-1α immobilized grafts at (**A**) 2 weeks and (**B**) 2 months after implantation. Nuclei were stained with Hoechst 33342 (**blue**). Dashed lines are used to distinguish the boundaries between L (lumen), N (neointima), G (graft), and T (surrounding tissues). Arrow indicates neovascularization on the outer surface of the grafts. Scale bar = 50 μm.

At 2 weeks after implantation, within the graft walls of both the heparin and heparin-SDF-1α groups, sparse cells that were αSMA^+^ but CNN1^−^ were observed near the luminal surface of the grafts (Figure 8A). The marker expression of these cells suggested they were in the early stages of differentiation into mature SMCs. In addition to the region within the vascular grafts, many αSMA^+^ and CNN1^+^ cells were recruited to the tissue surrounding the grafts in both the heparin and heparin-SDF-1α groups. The αSMA^+^ and CNN1^+^ cells around the heparin grafts were less organized, aligned in random orientation. In contrast, the αSMA^+^ and CNN1^+^ cells near the heparin-SDF-1α graft were organized and aligned in the same direction, forming a fibrous tissue structure which tended to provide more mechanical support.

At 2 months after implantation, both the heparin and heparin-SDF-1α grafts had developed a neointima. The heparinized grafts had developed neointimal hyperplasia. Most cells in the neointima were found to be αSMA^+^; only a small subset of these cells was also CNN1^+^ (Figure 8B). In comparison, no apparent lumen narrowing was observed in heparin-SDF-1α grafts, and there was only a thinner layer of normal neointima. The cells in the thin layer of neointima exhibited an organized and aligned structure, and were αSMA^+^ and CNN1^+^, suggesting that the cells are more differentiated into smooth muscle lineage. Cells in the tissue surrounding the heparinized graft and the heparin-SDF-1α grafts remained as αSMA^+^ and CNN1^+^, and these cells were organized and aligned, and begun to form a fibrous tissue structure. Both the marker expression profile and the orientation of the infiltrated SMCs showed that the heparin-SDF-1α grafts were better remodeled in comparison to the heparinized grafts.

### 3.10. Inflammatory Responses and the Recruitment of Macrophages

**Figure 9:**
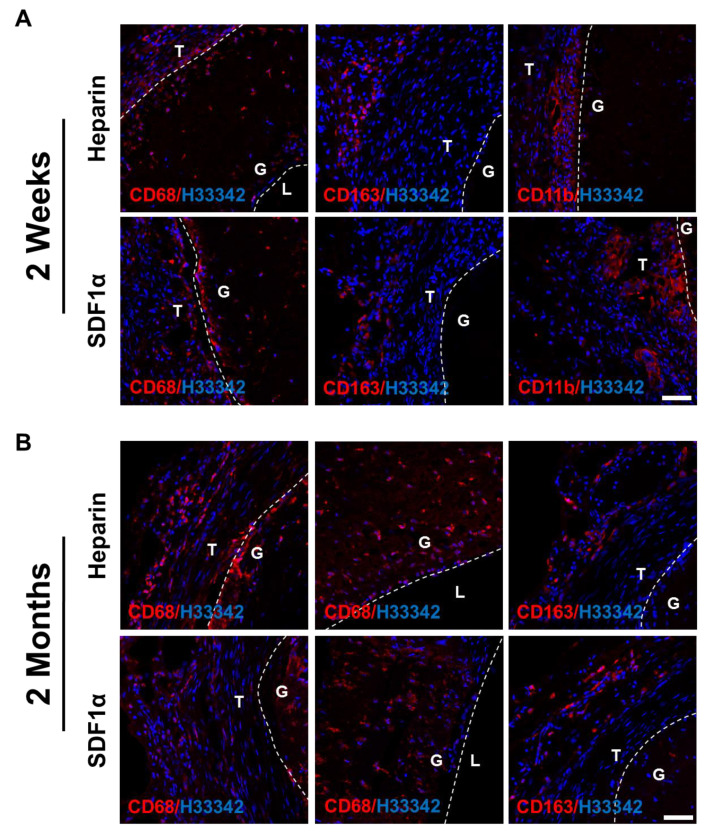
**Inflammatory responses of vascular grafts.** Immunostaining for CD68 (**red**), CD163 (**red**) and CD11b (**red**) of heparinized and SDF-1α immobilized grafts at (**A**) 2 weeks and (**B**) 2 months after implantation. Nuclei were stained with Hoechst 33342 (**blue**). Dashed lines are used to distinguish the boundaries between L (lumen), G (graft), and T (surrounding tissues). Scale bar = 50 μm.

Immune cells were also recruited to the vascular grafts since the grafts were foreign materials and would trigger inflammatory responses. To investigate the type of immune cells recruited, cross-sections of the grafts were immunostained for CD68, a pan-macrophage marker, CD163, an anti-inflammatory M2 macrophage marker, and CD11b, a monocyte marker. Overall, only a small number of CD68^+^ cells were found in all samples, mostly in the surrounding tissues near the outer surface of the grafts. The inflammatory responses and cell recruitment to the grafts did not exhibit a significant difference between the heparin and heparin-SDF-1α groups, at either 2 weeks or 2 months after implantation. At 2 weeks, anti-inflammatory CD163^+^ M2 macrophages were observed in the outer tissues surrounding the grafts (Figure 9A), suggesting that a remodeling mechanism was initiated by the host to repair the wound. In addition, CD11b^+^ monocytes were observed in the tissue directly adjacent to the grafts, marking the early stage of inflammatory responses. At 2 months after implantation, the CD11b^+^ cells disappeared (data not shown), and there were more CD68^+^/CD163^+^ cells in the grafts (Figure 9B), suggesting that the inflammatory responses had transitioned to a remodeling stage.

### 3.11. EPC Recruitment and Endothelialization

Endothelialization provides vascular grafts with the antithrombotic property, which was necessary for a graft to stay patent. To identify the cells lining the luminal surfaces of the grafts, *en face* and cross-section immunofluorescent staining against EC marker CD31 and EPC marker CD34 was performed on both the heparin and the heparin-SDF-1α groups. Hoechst staining of the cross-section of 2-week samples from both groups showed that more cells were observed within the grafts near the anastomotic ends than the mid-graft region. In addition, no noticeable difference in cell number was observed between the distal and the proximal end of the vascular graft (data not shown). This suggested that cells within the grafts near the anastomotic ends might have migrated from adjacent native carotid arteries. 

**Figure 10:**
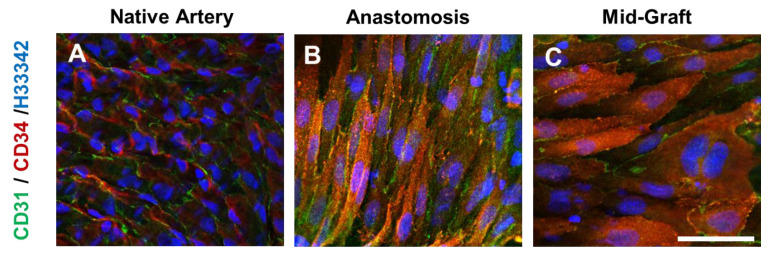
*En face* immunostaining for CD 31 (**green**) and CD34 (**red**) of the (**A**) native artery, and heparinized vascular grafts at 2 weeks after implantation at the (**B**) anastomotic and (**C**) mid-graft sites. Scale bar = 50 μm.

**Figure 11:**
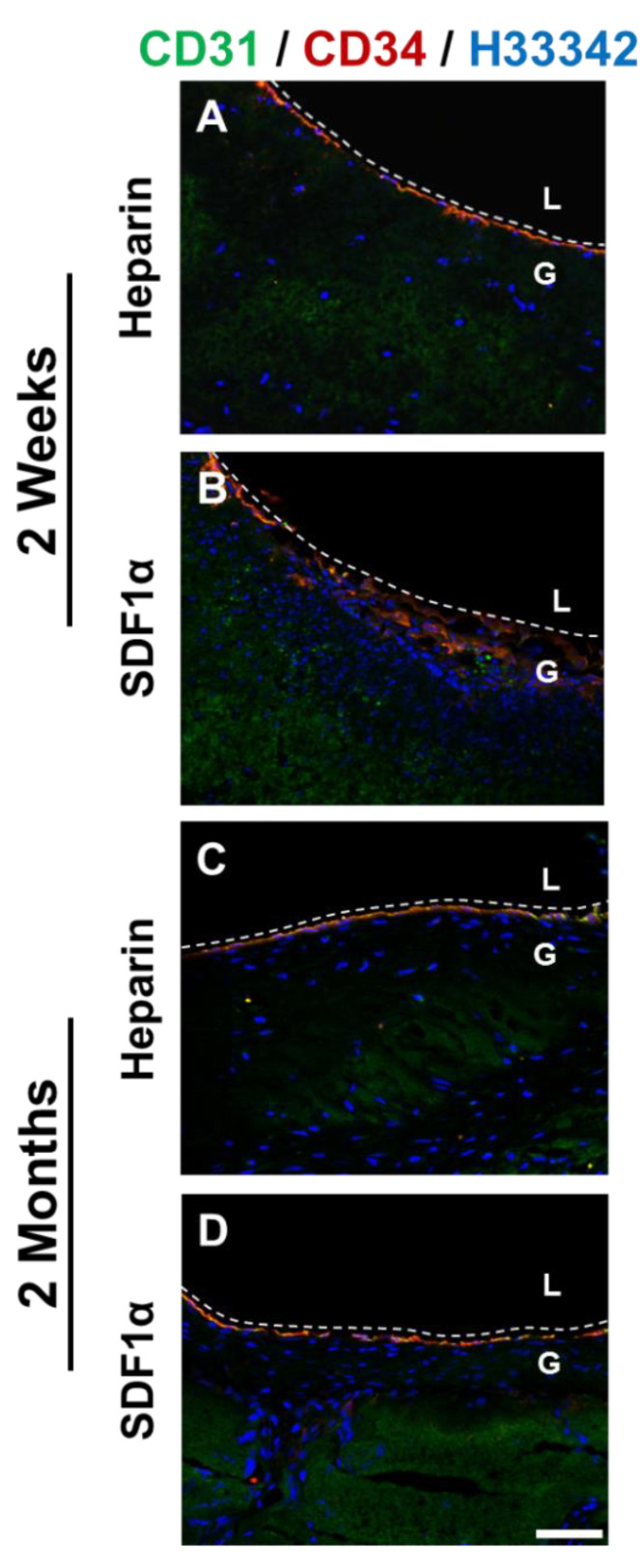
Immunostaining for CD 31 (**green**) and CD34 (**red**) of the cross-section of heparinized and SDF-1α immobilized grafts at (**A, B**) 2 weeks and (**C, D**) 2 months after implantation. Nuclei were stained with Hoechst 33342 (**blue**). Dashed lines are used to distinguish the boundaries between L (lumen), and G (graft). Scale bar = 50 μm.

**Figure 12:**
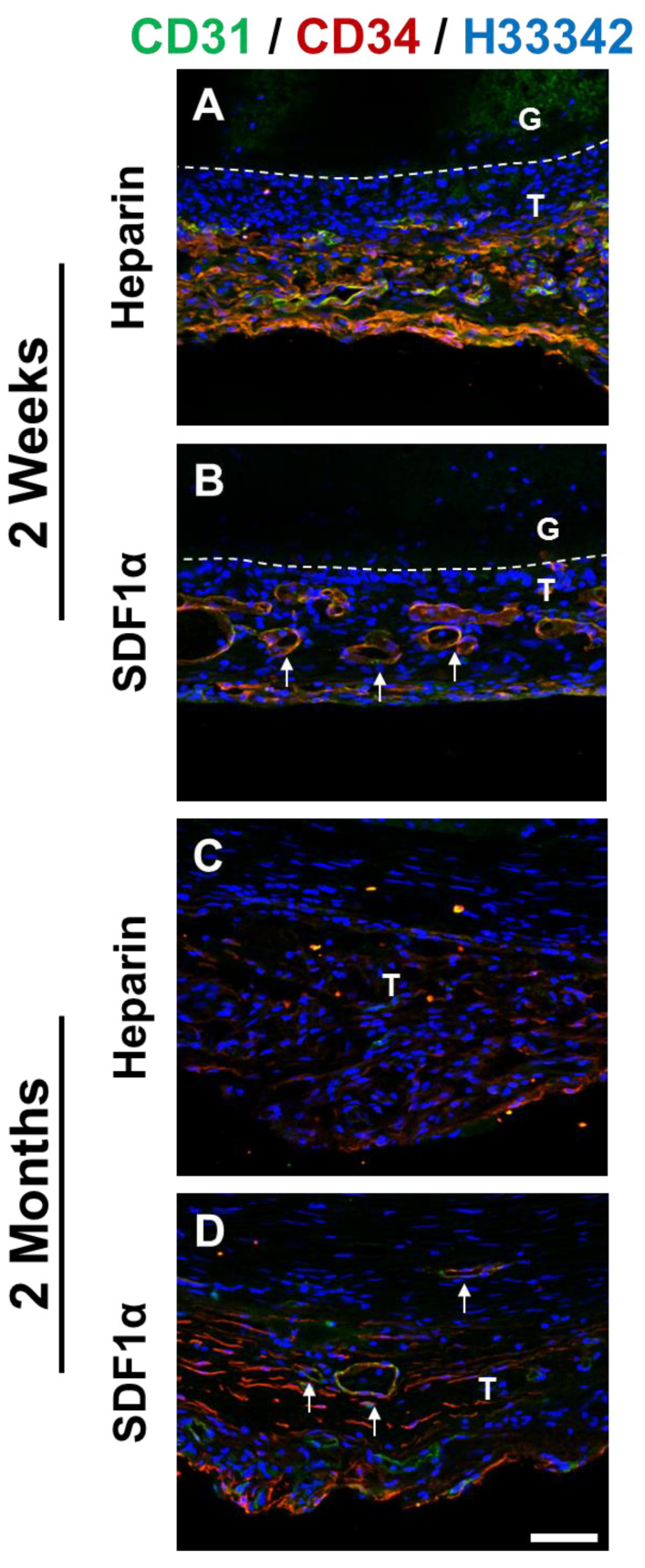
Identification and characterization of ECs and EPCs. Immunostaining for CD 31 (**green**) and CD34 (**red**) of the cross-section of heparinized and SDF-1α immobilized grafts at (**A**,**B**) 2 weeks and (**C**,**D**) 2 months after implantation. Nuclei were stained with Hoechst 33342 (**blue**). Dashed lines are used to distinguish the boundaries between G (graft), and T (surrounding tissues). Arrows indicate neovascularization on the outer surface of the grafts. Scale bar = 50 μm.

*En face* staining of heparinized grafts at 2 weeks showed that cells expressing both CD31 and CD34 were observed near anastomotic ends of the grafts (Figure 10). Although the cells had a well-defined cell–cell boundary, the cells were not aligned in the direction of blood flow as was the case in native blood vessels, suggesting that these cells were still in the process of remodeling. In addition to anastomotic ends, patches of CD31^+^/CD34^+^ cells were found throughout the grafts, suggesting a recruitment of these cells from the circulating bloodstream. Cross-section staining of 2-week graft samples showed the presence of ECs lining the luminal surfaces in both the heparin and the heparin-SDF-1α groups (Figure 11). In addition, in the tissue surrounding the grafts, more microvasculature structures were observed in the heparin-SDF-1α group, demonstrating the angiogenetic ability of SDF-1α (Figure 12).

At 2 months after implantation, the luminal surfaces of all patent grafts were almost completely covered by cells, including the regions that experienced partial degradation. Cross-section immunofluorescent staining showed CD31^+^ and/or CD34^+^ cells on the lumen of both the heparin and the heparin-SDF-1α groups (Figure 11). In the tissue surrounding the heparinized grafts, few CD31^+^ cells were detected. In contrast, in the tissue around the heparin-SDF-1α grafts, a greater number of cells had strong CD34 expression. In addition, CD31^+^ cells were observed in the microvessels in the surrounding tissue (Figure 12), further demonstrating the ability of SDF-1α in promoting angiogenesis and blood vessel maturation.

## 4. Discussion

Tissue engineering is a field that utilizes a combination of cells, materials, and engineering tools to improve or replace native tissues. A cell-free approach relies on the regenerative capability of the host. In this study, we developed a cell-free small-diameter microfibrous vascular graft with enhanced cell infiltration, characterized its biochemical and mechanical properties, and investigated the in vivo biological performance.

Previously developed methods to increase graft pore size/fiber spacing involve using post-electrospinning modifications such as salt leaching and using sacrificial fibers. The principles of both methods are similar. The salt particles or sacrificial polymer are temporarily electrospun into the grafts with the base polymer. They are later removed using water or appropriate solvents, leaving behind empty spaces. However, one major drawback of these two methods is that the base polymer would collapse and partially fill the empty spaces [29]. We therefore intended to fabricate vascular grafts with larger pore size/fiber spacing without any post-electrospinning modification by blending a fast-degrading polymer. A fast-degrading polymer would shorten host exposure to the foreign materials and allow rapid host remodeling. For instance, researchers have developed a vascular graft with polycaprolactone (PCL) and a fast-degrading elastomer poly(glycerol sebacate) (PGS) [30]. The graft consists of a PGS core that was fabricated by means of a salt fusion and leaching method, and a PCL sheath that was electrospun onto the PGS core. The graft degrades rapidly in vivo and allows the formation of neoarteries that are nearly free of foreign materials in 3 months. Although PGS is a promising candidate, it is difficult to work with technically. PGS requires gentle handling due to its low tensile strength. Moreover, electrospinning PGS is challenging because crosslinked PGS is insoluble in the organic solvent, which makes the use of uncrosslinked PGS prepolymer necessary. However, the glass transition temperature of PGS prepolymer is below room temperature, which causes the polymer to flow and electrospun fibers to fuse into a nonporous structure at room temperature. This fiber fusion is aggravated by the high temperature required for thermal crosslinking [31]. Given these technical challenges, in this study, we used a different fast-degrading polymer, PDO. In addition to its rapid degradation rate, the intrinsic properties make PDO a great candidate to enhance cell infiltration. Comparing to PLCL under the same conditions, PDO fibers are less tightly packed and have higher porosity, potentially due to its weak intramolecular adhesion caused by its intrinsic chemical property. Moreover, it is easy to handle and compatible with electrospinning, and is readily dissolvable in Tris-HFIP (the optimized solvent for PLCL according to laboratory protocols). Typically, faster degrading materials trigger more pronounced inflammatory reaction than their slower degrading counterparts [32,33], indicating that a balance between the cell infiltration and degradation rates is mandatory. In this study, using PLCL and PDO and the electrospinning technique, we developed a PLCL/PDO blend graft that reached a good balance between the cell infiltration and degradation rates.

In addition, the PLCL/PDO blend grafts had an elastic modulus on the same order as the native artery. The grafts were able to meet the most fundamental physical requirement of withstanding the suture force during anastomosis surgery and arterial pressure immediately upon implantation, as well as staying in shape and providing structural support after 2 months of implantation under in vivo environment, in which the degradation and loss of mechanical strength were accelerated due to hydrolytic as well as enzymatic degradation [34]. This suggests that both the cells infiltrated into the grafts and the tissue growing around the grafts compensated for the loss of mechanical strength.

In addition to providing mechanical and structural supports, an ideal cell-free vascular graft should be able to tap into the full regenerative potential of the host to promote endothelialization and remodeling of the vascular wall. Acute thrombosis is the major reason for failure in small-diameter vascular grafts. Although it can be temporarily suppressed by heparin, an anticoagulant with anti-thrombogenic property, oral anticoagulant and antiplatelet therapies might still be necessary to maintain long-term patency. Endothelium produces anticoagulants such as heparin sulfate and, hence, provides a non-thrombogenic surface. Therefore, for a vascular graft to succeed, it is crucial to have a rapid endothelialization of the graft. SDF-1α is a chemokine that has been reported to attract EPCs and accelerate endothelialization of vascular grafts [16]. However, SDF-1α is small in size (~10 kDa), which would lead to a fast diffusion when it is passively absorbed onto the grafts. Moreover, the active site of SDF-1α is cleavable, for instance, by proteases, dipeptidyl peptidase IV/CD26 and matrix metalloproteinase-2, which causes fast SDF-1α degradation in vivo [35]. Heparin has been reported to bind and stabilize SDF-1α without interfering with its chemotactic activities [20,21,22,28]. The *in vitro* results reported herein showed that immobilized SDF-1α on heparinized grafts was more stable and had slower *in vitro* release than passively absorbed SDF-1α. The in vivo studies showed that the vascular grafts were able to recruit ECs and EPCs within 2 weeks. However, partial coverage of the luminal surface by ECs and EPCs were observed throughout the grafts, suggesting that complete endothelialization takes more than 2 weeks to finish. There are two main mechanisms whereby vascular grafts recruit ECs. The first mechanism is the transanastomotic migration of ECs from the adjoining arteries. The second mechanism is adhering and differentiation of EPCs recruited from the bloodstream. SDF-1α immobilized on the vascular grafts is expected to recruit more EPCs from the bloodstream; it can further promote endothelialization of the grafts by inducing proliferation and differentiation of adhered ECs [36,37].

Other than endothelialization, another important criterion for a successful vascular graft is remodeling of the vascular wall as SMCs contribute to the necessary elasticity and mechanical strength of the vascular grafts. The results reported herein showed that the heparin-SDF-1α grafts were able to recruit SMCs, demonstrating that the host possesses the potential to carry out vascular regeneration with endogenous cells without using a cell-seeding graft. The advantage of cell-seeding grafts is that they can enhance biocompatibility and the anti-thrombogenic property, which might lead to better long-term patency. However, the approach faces various challenges such as cell characterization, survival in the grafts during preparation and surgery, and attachment under native flow condition.

In this study, we demonstrated the important roles of both the biophysical and biochemical properties of vascular grafts in blood vessel regeneration. This reported vascular graft engineering approach along with chemokine immobilized onto chemically modified surfaces could serve as a foundation to be further improved upon. The effect of SDF-1α was investigated using a healthy rat model, which has a strong regenerative ability and cannot fully recapitulate the response in human beings. Their ECs have a greater capacity to migrate and cover the luminal surface of the vascular grafts than those of humans, especially aged humans with diseased blood vessels [38,39]. The performance of heparin-SDF-1α grafts in large animals is yet to be investigated. It is expected that SDF-1α will have a more pronounced effect in large animals and possibly be an essential component in a disease model. Furthermore, the in vivo performance of the vascular grafts was studied up to 2 months after implantation; longer follow-up study is expected to reveal more informative data on critical aspects such as aneurysmal formation and late calcification. Although aneurysm formation was not observed in heparin-SDF-1α grafts in this study due to their adequate tissue integration, it is essential to examine whether the mechanical support provided by newly-formed neotissue remains adequate when the grafts are further degraded in the long-term study. Another long-term biological performance index is vascular graft calcification. The accumulation of macrophages in the foreign vascular graft could induce and influence the propagation of the inflammation-dependent calcification, which has a detrimental effect on long-term biological performance and may cause eventual graft failure [40,41,42,43]. No calcification was observed at 2 months after implantation in this study. To study how the degree and extent of calcification are associated with the duration of graft implantation, a long-term study would be required in order to conduct a comprehensive assessment.

The information gleaned from this study has provided much needed insight into the field of vascular regeneration and provides a path for future studies to tune and improve vascular graft technology. In addition, this advancement will not only lead to the acceleration of the functional vascularization, but also facilitate the development of novel scaffolds with broad applications in regenerative tissue engineering.

## Figures and Tables

**Table 1:** Antibody information for immunohistochemistry.

Antibody	Company	Catalog #	Dilution
CD31	Abcam	ab28364	1:50
CD34	R&D Systems	AF4117	1:40
αSMA	Abcam	ab5694	1:100
CNN1	Abcam	ab46794	1:100
CD68	AbDSerotec	MCA341R	1:100
CD163	Santa Cruz	sc-58965	1:50
CD11b	Abcam	ab8879	1:100

## Data Availability

The main data supporting the results in this study have been summarized and presented in the paper. Additional information can be obtained from the authors upon request.
